# It’s not my fault although it might be: chiropractic practice and vicarious liability

**DOI:** 10.1186/s12998-021-00379-0

**Published:** 2021-06-14

**Authors:** J. Keith Simpson, Stanley Innes

**Affiliations:** Discipline of Psychology, Exercise Science, Counselling and Chiropractic (PESCC), College of Science, Health, Engineering and Education (SHEE), Murdoch University, Murdoch University, Murdoch, Australia

**Keywords:** Duty of care, Negligence, Vicarious liability, Non-delegable duty of care, Chiropractic

## Abstract

**Background:**

While chiropractic care is most commonly provided within a private practice context, the ‘traditional’ solo practice is now uncommon. Chiropractors, manual therapists and related health professionals commonly work within the same practice bringing obvious advantages to both the practitioners and their patients. However, multi-practitioner, multi-disciplinary clinics also carry often unrecognized liabilities. We refer here to vicarious liability and non-delegable duties. Vicarious liability refers to the strict liability imposed on one person for the negligent acts of another person. The typical example is an employer being held vicariously liable to the negligent acts of an employee. However, vicarious liability can arise outside of the employer-employee relationship. For example, under non-delegable duty provisions, an entity owing a non-delegable duty can be liable for an independent contractor’s wrongdoing.

After a plain English explanation of this complex area of law, we provide seven scenarios to demonstrate how vicarious liability can envelop practice principals when things go wrong. We also make suggestions for risk mitigation.

**Conclusion:**

Practice owners may unexpectedly find themselves legally liable for another’s actions with dire consequences. A knowledge of vicarious liability along with implementing risk mitigation strategies has the potential to minimize the likelihood of this unwanted event. Recommendations are made to this end.

## Background

Negligence is a failure to take reasonable care to avoid causing injury or loss (harm) to another person. It is clearly something to be avoided. In our previous paper we discussed negligence in the context of informed consent [1]. Under certain circumstances, a health care professional (HCP) may find themselves liable for the negligent conduct of another service provider. This is because the relationship between the HCP and the other service provider is one in which the HCP has vicarious liability for harm caused by the actionable conduct of the other or because the HCP breached the HCP’s non-delegable duty of care. This is not a new concept. The Latin legal phrase, *qui facit per alium facit* per se, means “He who acts through another does the act himself.” With its origins in ancient Rome where masters were vicariously liable for the wrongdoings of their slaves, this maxim is now often stated when discussing the liability of employers for the negligent acts of employees in terms of vicarious liability. It is a fundamental legal axiom of the law of agency: A principal who appoints or authorises an agent to act for them will be bound by the acts of the agent in the performance of the principal’s authority [2]. *Qui facit per alium facit* per se is closely related to the Latin maxim – *respondeat superior*, which forms traditional basis of vicarious liability. *Respondeat superior * means that an employer is liable for the consequences of any act done by employees in the ordinary course of their duties and responsibilities [3].

There is a strong trend toward chiropractors working in multidisciplinary and integrative care settings. In the USA, over half of the chiropractors surveyed in 2020 reported that they operated in a multidisciplinary clinic compared to 29% in 2019 [4]. About a quarter responded that they work in an integrated practice--up significantly from 6% the previous year [4]. Adams et al. found that the 78% of surveyed Australian chiropractors work in a multi-practitioner setting and 46% work in a multi-disciplinary setting [5]. Without an understanding of duty of care, vicarious liability, and non-delegable duty of care – meaning the duty of care cannot be passed off to another person – and the need to take proper precautions to mitigate risk, it is conceivable that the HCP will find themselves defending a liability suit for the negligent actions of another.

With ever-changing labour markets and increasing litigiousness, this area of law is something of a minefield that keeps the legal profession busy [2, 6–8]. Some form of a doctrine of vicarious liability, also known as ‘liability for the acts of others’, exists in the legal system of all Western countries. Indeed, there are universalist implications of this discussion. This is because, in all existing legal systems, there are strong similarities in the legal uses of the vicarious liability or *respondeat superior* doctrine whereby the superior is responsible for the acts of their subordinates. Even though there are jurisdictional variances [9] none make a difference to the application of the doctrine in the health care setting [10]. Thus, the principles used to explain the seven scenarios below apply in all Western countries under their respective vicarious liability provisions.

In 1916 Laski wrote of vicarious liability “in no area of legal thought are the principles in such sad confusion [11] p.105. One hundred years later Susan Kiefel, Chief Justice of the Supreme Court of Australia, pointed out that vicarious liability remains an anomaly in the law of torts. Vicarious liability requires *A* to pay *B* because *C*, acting as *A’s agent,* has injured *B*, where *A* was not personally at fault [12]. Unless *A* works as a completely solo practitioner, never engaging a *C*, a basic understanding of vicarious liability is important.

This paper explores vicarious liability, the duty of care in negligence and liability for breach of the duty of care. We do this by examining a variety of practice arrangements in seven scenarios where vicarious liability and non-delegable duties come into play. To assist practitioners, we offer recommendations to mitigate this type of liability exposure.

### Duty of care

The duty of care that the HCP owes the patient is a fundamental principle of health care practice [13]. It signifies the HCP’s obligation to provide quality health care and, for the conscientious HCP, the commitment to honour that obligation [14]. The scope of the obligations surrounding a HCP-patient interaction within the confines of a standard health care professional –patient relationship is relatively settled in Australia and elsewhere; HCPs have a legal obligation to patients to adhere to a standard of reasonable care [15] unless the law imposes a higher standard in the circumstances or the HCP has agreed to a higher standard.

For chiropractors, the standards are set out in the regulatory body’s code of conduct and any professional association’s code of ethics as supplemented by the common law or other legislation. In essence, the standards delineate the scope of the duty of care and the standard of care in the ordinary case and what must be done to fulfil that duty. In addition, in a particular case, an HCP may have agreed to an added requirement which would not apply but for the agreement. The HCP may always increase the standard of care with or without the client’s consent. In general, and in practice, the HCP cannot reduce the standard of care, even with the patient’s consent. There are instances in which the HCP will have a valid reason for conduct otherwise below the standard of care. For example, administering treatment to an unconscious patient in an emergency situation without consent would not be considered a breach of the standard [16].

In Anglo-American jurisprudence, the common law (case-based law) surrounding the duty of care in the absence of a contract was first formally recognized in 1884 in *Heaven v Pender* (UK):

If a person contracts with another to use ordinary care or skill towards him or his property, the obligation need not be considered in the light of a duty; it is an obligation of contract. It is undoubted, however, that there may be the obligation of such a duty from one person to another, although there is no contract between them with regard to such duty [17].

It wasn’t until 1932 that the landmark case of *Donoghue v. Stevenson (UK)* clarified the basis of the duty of care in the tort of negligence. It established the ‘*neighbour principle’* and obliged all people to observe a duty of care towards their ‘neighbours’. For Commonwealth countries, *Donoghue v Stevenson*, establishes that there is a general duty to take reasonable care to avoid foreseeable injury to a ‘*neighbour’* [18]. The principle had been established in the United States about 1 ½ decades earlier.

Torts are civil wrongs resulting in harm. The tort of negligence is part of the law of torts. Under the tort of negligence, an individual’s duty of care is to not injure another person (*neighbour*) by their negligence. Any person who acts in a manner that foreseeably results in another’s injury or loss (harm) will be liable in negligence for that harm if the requirements of the cause of action are met. There is no tort if there is no harm [19]. Under the tort of negligence, the individual’s duty of care is to not injure through carelessness – that is, through negligence – a person who qualifies as a *neighbour*. One’s *neighbour* is any person who may foreseeably suffer injury because of one’s negligence. Any person who acts negligently in a manner that foreseeably results in another’s harm will be liable in negligence for that harm if all the other requirements for actionability are met.

### Establishing negligence

Establishing negligence requires four criteria to be satisfied.

(i) Legally recognized harm
(ii) The harmed person is owed a duty of care.
(iii) A breach of a duty of care is established, and
(iv) Legally recognized harm has been caused by the breach [20].

In the health care setting, the first and second requirements are easily established. Physiological injury, psychological injury, and financial loss are examples of legally recognized harms. There is obviously a duty of care in place with existing patients. Also, a duty of care may exist between HCPs and a prospective patient once an appointment has been made even if they have not attended the practitioner [21], and between medical administrators and hospital patients [22]. The person making the claim (Claimant) must establish, on the balance or probabilities:

- that a breach of a required standard has occurred;
- the harm was reasonably foreseeable; and in most cases,
- the harm was caused by the negligent conduct.

In most cases the claimant establishes causation – the causal relationship between the defendant’s conduct and the harm – by satisfying some version of a ‘*but-for’* test. The *but-for* test says that the harm would not have occurred *but-for* the negligent conduct of the HCP (defendant) [23]. If the *but-for* test is satisfied, compensation is paid to return the claimant to the position in which they would theoretically have been had the harm not occurred. Western legal systems generally recognize that the *but-for* test is inadequate. This is because in most instances the harm has been caused by more than one independently necessary and sufficient condition [24]. That is, in these cases, there is more than one *but-for* event. Causation in law is a complex and controversial subject [19]. The law has devised rules for resolving causation in ways generally considered satisfactory and fair [25, 26]. Additional discussion of causation issues is beyond the purpose and scope of this paper.

There are added dimensions worth mentioning briefly, because of the fiduciary aspect of the HCP-patient relationship. Goldberg refers to these dimensions as the fiduciary duty of care [27]. Unlike a breach of tort law, the breach of a fiduciary duty of care is not exclusively a duty to not cause harm [27]. This means a breach of fiduciary duty of care can generate accountability, even if the breach does not result in injury. Specifically, the duty of care owed by a fiduciary to a beneficiary is a duty of judicious conduct and proper performance unconditionally rather than a duty to avoid causing injury through legally wrongful conduct [27]. For example, failure to obtain valid informed consent or keep proper clinical notes are breaches of professional standards. This constitutes professional negligence and may attract administrative penalties affecting the HCP’s ability to practice legally even if there is no harm to the patient.

### Practice scenarios

We present seven health care scenarios for consideration. Scenarios one through six are hypothetical HCP/patient relationships under which a potential liability emerged. We then analyse the scenarios to determine the likelihood of how liability may be decided. The explanations are based on rulings in vicarious liability cases from various jurisdictions. Scenario seven presents an actual vicarious liability case - *Alexander v Heise -* involving an HCP in New South Wales, Australia. It is illustrative of how VL law is applied. While this is an Australian case, the facts are not unique to Australia and the outcome of the case would likely be the same in all jurisdictions with VL provisions.

### Definitions

Because of the global intent of this paper, some terms used may not be universal. Terms used in the health care scenarios are defined as follows. The definitions will likely match titles used in other jurisdictions.

Principal: The owner(s) of a business.

Associate: Any person ‘associated’ with the business other than a principal. Depending on the contractual arrangements this may be an employee, a contractor, a locum, or a tenant.

Employee: An employee is an individual hired by an employer to do a specific job in what is intended to be (or the law deems to be) an employer-employee relationship. An employee, working under a ‘contract of service’ or ‘contract of employment’, must submit to the orders and instructions of his employer [28]. They receive employee benefits such as paid leave, workers compensation coverage etc.

Contractor: A contractor (independent contractor), acting under a ‘contract for services’, is intended to possess greater autonomy than an employee [28]. They have a high degree of control over how and when the work is done. They may, but are not required to, supply their own tools and equipment. They do not receive employee benefits.

Locum Tenens: An HCP working temporarily in another practice, not their own. A locum tenens may be an employee or contractor depending on the ‘contract for services’. [29].

Partnership: A partnership is a formal arrangement by two or more parties to manage and operate a business and share its expenses and profits. Generally, partners are jointly liable to persons with whom the partnership, or any of the partners, deal with partnership matters, regardless of anything in the partnership contract.

Tenant: A person or group (lessee) that rents and uses a serviced office, from another (lessor) for a period of time under the terms of a rental agreement (lease). The lessee operates their business separately from any other lessee similarly renting office space.

### Scenario 1: A solo practitioner and a patient

Patient *C* presented to *Dr D* with low back pain. *Dr D* failed to recognize a prodromal disc presentation, manipulated *C’s* lumbar spine resulting in a frank disc herniation which required surgery. Is Dr. *D* liable in negligence? Answer: Yes.

### Explanation

The HPC is solely responsible for their work and thus has liability for their own negligent actions. Application of the negligence test confirms liability: Legally recognized harm was caused to a person owed a duty of care by the HCP’s breach of their duty of care.

### Scenario 2: A practice associate (acupuncturist) and a patient

Patient *C* presents to *Dr D’s* clinic where they receive acupuncture administered by associate Acupuncturist *A*. *A* is *Dr D’s* agent retained under a written agreement. During the course of treatment, an acupuncture needle breaks leaving a fragment lodged in *C*. Is *A* liable in negligence? Answer: yes, *A* is responsible for their own negligent action. Is *Dr D* also liable? Answer: Possibly. Here the agreement is in writing, but the result would be the same if it was not.

### Explanation

The issue is whether the acupuncturist is or is not the HCP’s employee. Employers are vicariously liable for the negligent actions of their employees which occur in the scope of their work. This may include acts of discrimination, defamation or harassment occurring in the workplace or in connection with a person’s employment [30]. Further, an employer may be held vicariously liable even if the employee acts in direct contradiction of their employer’s instructions or prohibitions [10].

Vicarious liability (VL) is the imposition, by law, of legal and hence usually financial responsibility on someone, typically an employer, for the harm and damages that result from the actions of another person, typically their employee, even though the employer has done nothing wrong. The employer’s vicarious liability is in addition to the liability of the tortfeasor (wrongdoer). Additionally, if the employer was negligent in hiring the employee, and that negligence is relevant to the manner in which the harm was caused, then the employer’s own conduct is also a cause of the harm. This would be an instance of employer breach of duty of care in hiring a competent employee.

In all jurisdictions in which this paper applies, the principles underpinning the application of VL are: a solvent defendant, encouraging employer vigilance and distributive justice [31]. The rationale for VL is that the consequences of the employee’s errors should be borne by the employer thereby increasing the likelihood there will be a person with sufficient assets to compensate the injured person. This allows the injured party (plaintiff) the opportunity to access compensation from the *respondeat superior* or, coarsely expressed, *deep-pocket defendants* typically with assets greater than the tortfeasor [32]. It is important to understand that VL is strict liability which makes people pay compensation for damages even if they are not at fault. Specifically, the vicariously liable party has committed no breach of duty to the plaintiff but is held legally answerable because of the legal imposition of legal responsibility for another’s fault; without the need of finding of fault (such as negligence or tortious intent). The claimant need only prove that the tort occurred and the existence of the vicarious liability relationship [30].

A ruling of VL typically hinges on the employer/employee conditions contained in the contract of employment or as imposed by law [28]. The claimant will argue that a master/servant (employer/employee) relationship [2] existed between *Dr D* and *A* and hence VL is available as the basis for the employer’s liability. The defendant will argue that the actual wrongdoer was retained on some basis that did not create a vicarious liability relationship; that is as an independent contractor.

In the past the courts focussed on the extent to which the employee was integrated into the business. Today, the courts will examine the entire relationship between the parties, including the terms of any formal or informal agreement if any between the ‘employer and the alleged employee’ to decide whether an employer/employee or employer/independent contractor relationship existed. Table 1, derived from recent Australian court cases [33, 34], demonstrates the principal indicators used in determining whether a person is likely to be an independent contractor.

**Table 1:** Independent contractor or employee?

Indicator	Employee	Independent contractor
Degree of control over work	Performs work, under the or right of direction and control of their employer, on an ongoing basis	Has a high level of control in how the work is done
Other employers.	Employee works solely for the employer. [This generally refers to full-time employment – some employees may choose to work additional jobs].	Worker performs work for others or is genuinely entitled to do so.
Skill	The work does not involve a profession, trade or distinct calling on the part of the employee.	The work involves a profession, trade or distinct calling on the part of the worker.
Equipment	Employer provides and maintains significant tools or equipment.	Worker provides and maintains significant tools or equipment.
Hours of work	Generally, works standard, set hours set by the employer with an ongoing expectation of continued work.	Under agreement, the worker decides what hours to work to complete the specific task.
Payment	Employee is paid by periodic wage or salary.	Worker provides invoices after the completion of tasks.
Tax	Employer deducts income tax from remuneration paid.	Worker responsible for own tax affairs.
Benefits	Employer provides paid holidays or sick leave to workers.	Worker does not receive paid holidays or sick leave.
Termination	Can be discharged at any time for ‘good cause’. Good cause can include things like poor work performance, violating company rules and threats of violence.	May be terminated subject to the sub-standard or non-performance clauses in their contract.
Unfair dismissal claim.	Can lodge an unfair dismissal claim.	Cannot lodge an unfair dismissal claim.
Asset creation	The worker of creates goodwill or saleable assets for the employer’s business.	The worker creates goodwill or saleable assets for their own business.
Business expenses	The employee does not spend a significant portion of their pay on business expenses.	The worker spends a significant portion of their remuneration on business expenses.

Source: Australian Fair Work Commission. https://www.fwc.gov.au/unfair-dismissals-benchbook/coverage/people-excluded/independent-contractors#_ftn17

The table above is not exhaustive and whether a worker is an employee or contractor may be determined by a factor other than those listed

Whilst the designation of a person in their contract as well as their job description will provide evidence of the person’s status, it will not provide conclusive evidence. Having assessed the above factors in Table 1, the court will consider a crucial factor in determining whether there is an employment relationship or an independent contractor relationship: the right of the employer to control the work of the agent [35]. Some analysts have extended this concept to include incentivised contractor agreements. For example, an agreement that increases remuneration based on reaching performance targets is more suggestive of an employer/employee relationship and with it employer liability for contractor negligence [36].

Today, the courts acknowledge these tests cannot definitively determine whether or not a person is an employee, although they may still factor into the court’s deliberations. The courts will look at all the facts of the case when determining whether a person is an employee, paying particular attention to apparent agency, control test, and non-delegable duty of the principal for patient care [37].

As a rule of thumb, if the employer has the right to control the work, an employer/employee relationship exists, whereas, if the employer has no right to control the work, an independent contractor relationship exists. The critical consideration is the right of the employer to exercise control, regardless of the actual exercise of the control [35]. If *Dr. D* has no right to control *A’s* work, *Dr. D* may not be vicariously liable but may face liability for non-delegable duty of care (see Example 5 below). More information regarding the agreement between *Dr. D* and *A* is required to determine *Dr. D’s* liability in the example given.

### Scenario 3: A practice ‘associate chiropractor’ and a patient

Patient *C* presented to *Dr D’s* clinic with low back pain. *C* is attended by *Dr A*, who is *Dr D’s* ‘associate chiropractor’. *Dr A* failed to recognize a prodromal disc presentation, manipulated *C’s* lumbar spine resulting in a frank disc herniation which required surgery. Is *Dr A* liable in negligence? Answer: Yes. Is *Dr D* liable? Answer: Possibly. Depending on the facts, *Dr D* may be liable in VL for *Dr A’s* action, breach of non-delegable duty for *Dr A’s* actions or breach of *Dr D’s* own duty of care.

### Explanation

*Dr A* is liable for their own negligent actions. In the absence of any legal requirement, *Dr D* is under no obligation to review *Dr A’s* cases. As discussed in Example 2, the nature of the ‘contract of service’ between *Drs D* & *A* will affect *Dr D’s* potential liabilities. *Dr D* may be vicariously liable if *Dr A* is an employee. For example, if *Dr D* had the right to control the work of *Dr A*, then *Dr A* is considered an employee and *Dr D* will be vicariously liable provided the task resulting in the injury was within the ‘course and scope’ of the employment, even if the right to control is minimally exercised. Let’s say *Dr D* ‘reviews’ *Dr A’s* cases and approves care plans. This would be considered exercising the right to control, even though *Dr A* administers all care. *Dr D* is vicariously liable. On the other hand, if *Dr D* plays no part in *Dr A’s* actual patient care, and there is no provision for such a role in their arrangement, *Dr D* will not face a vicarious liability case.

*Dr D* & *Dr A* may face another legal problem. If the practice does not comply with the laws and regulations as set out in the regulatory body’s code of conduct and other applicable legislation, they both may be in breach of their duty of care for non-compliance with the standards resulting in the imposition of disciplinary penalties. For example, if *Dr D’s* practice is to x-ray every new patient before placing them on extended care plans without valid informed consent, *Dr D’s* practice would not comply with the code of conduct in Australia. If *Dr A*, as a condition of their engagement, is required to follow and follows *Dr D’s* practices, both would be in breach of their duty care to comply with the required standards and face administrative penalties affecting their ability to practice.

*Dr D* may still have a legal problem if it can be shown they did not take all reasonable steps to see that *Dr A* was a competent practitioner before engaging *A’s* services and to fulfil *Dr D’s* duty to provide safe equipment and safe premises [38]. Failure to do so would bring allegations of a breach of a duty of care on *Dr D’s* part. The particular problem that results will determine whether the conduct of *Dr A* is the breach – therefore a non-delegable duty instance – or the conduct of *Dr D* that is the breach, in which case there is an ordinary breach of duty action.

### Scenario 4: Practice associates see a patient

Patient *C* presented to *Dr D’s* clinic with low back pain. During one office visit, *C* is attended by *Dr L*, who is *Dr D’s* ‘locum chiropractor’. *C* is also treated by *A* the clinic acupuncturist and *M* the clinic massage therapist. *Dr L* failed to recognize a prodromal disc presentation, manipulated *C’s* lumbar spine resulting in a frank disc herniation which required surgery. *A* inserted an acupuncture needle that punctured *C’s* lung while *M* failed to recognize that *C* was on anti-coagulants and *C* sustained extensive painful bruising following the massage therapy. Are *Dr L*, acupuncturist *A* and massage therapist *M* liable in negligence? Answer: Yes. All are liable. Is *Dr D* liable? Answer: possibly in VL or a breach of *Dr D’s* own duty of care.

### Explanation

*Dr L*, acupuncturist *A* and massage therapist *M* had common purpose – treating *C’s* low back pain – and are liable as joint tortfeasors. Their collective negligence in caring for *C* caused *C* harm. This made them jointly responsible for the compensation in money (damages) as imposed by law for *C’s* loss or injury. The amount of the damages paid by each tortfeasor will be determined by case law or legislation in the particular jurisdiction where the harm occurred [39, 40].

Depending on the contracts of employment with each, *Dr D* may be vicariously liable or liable for breach of duty of care. In any event, *Dr D* may be liable based on the breach by the others of *Dr D’s* non-delegable duty (see scenario 5).

### Scenario 5: A patient and a clinic operated by a national chain of clinics

Patient *C* presented with low back pain to Everywhere Chiropractic Clinic (ECC) in *C’s* suburb. ECC is part of a national chain of chiropractic clinics operating in many capital cities. ECC advertises their high-quality care. *Dr D* is one of numerous chiropractors retained by ECC across the country. *Dr D* provides *C’s* care. *Dr D* failed to recognize a prodromal disc presentation, manipulated *C’s* lumbar spine resulting in a frank disc herniation which required surgery. Is *Dr D* liable in negligence? Answer: Yes. Is ECC liable? Answer: possibly for breach of a non-delegable duty of care.

### Explanation

*Dr D* is liable for their own negligent actions. ECC had a non-delegable duty of care to those attending ECC clinics in relation to the competence of *Dr D* and thus may be vicariously liable for harm resulting from *Dr D’s* negligence. ECC’s duty of care includes establishing a proper system of care, a duty to engage competent staff and a duty to provide proper and safe equipment and safe premises [10]. These duties cannot be ‘delegated’ to another in the sense that the delegation relieves ECC of responsibility for the ‘breach’; as such, breach of any of them by the person delegated to act may attract liability.

Liability based on the breach of a non-delegable duty (NDD) is a form of strict liability. This is because it is not based on one’s own conduct being the breach of the duty. It is a duty which cannot be assigned to someone else, hence the term, non-delegable duty. NDD is used to justify the imposition of liability on one person or entity for the negligence of another to whom the entity has entrusted the performance of some task on their behalf. NDD developed as a legal workaround because of the limitations of the doctrine of vicarious liability. In essence NDD is a mechanism allowing the imposition of what amounts to VL when the negligent act is committed by an ‘independent contractor’ [7]. In practice this means that when a person owes a non-delegable duty towards another, the person has a duty not only to take reasonable care themselves but also must take reasonable care to see that others take reasonable care in order to avoid breaching the duty themselves. In addition, the person may also be liable even if they have done all of that, but the others still do not take reasonable care in the performance of a delegated action, so the conduct of the others is the breach of the NDD. Typical examples of forms of the NDD can include the duty of care hospitals have to their patients regarding the competence of the HCPs provided to care for patients and that schools have to their students when engaging independent contractors to provide services such as swimming lessons [7].

In the scenario, under the doctrine of non-delegable duty, the ECC may not pass off to another (any of its associate chiropractors), the duty to take reasonable care that it would have if it provided the service or did the work itself, merely by retaining a person competent to provide the service or perform the work. Retaining the person does not relieve ECC of the duty of care [41]. Engagement of independent contractors (and “delegation” of the relevant activity to them) remains permissible and is a common, generally necessary practice. However, to avoid breaching their aspect of the NDD themselves, the ECC must see that they exercise due diligence in engaging the contractor and must see that reasonable care is taken by contractors and associates to whom they have entrusted the care of the claimant [41]. In the example, if the work was done negligently, ECC will face NDD based liability for *Dr D’s* negligence.

### Scenario 6: A tenant HCP and a patient

Patient *C* presented to *Dr T* with low back pain. *Dr T* failed to recognize a prodromal disc presentation, manipulated *C’s* lumbar spine resulting in a frank disc herniation which required surgery. *Dr T* leases serviced clinic rooms from landlord, *Dr L*. Is *Dr T* liable in negligence? Answer: Yes. Is *Dr L* also liable? Answer: Probably no but maybe yes.

### Explanation

*Dr T* is responsible for their own negligent action. *Dr T* is a tenant in leased office space. *Dr L* faces no liability unless it can be demonstrated that *Dr L* breached the contractual terms of the lease, for example failing to provide safe premises in a way relevant to the injury that occurred or *Dr L* was consulted on the case which somehow contributed to the harm to *C*. To avoid potentially misleading patients, *Dr T* would be wise to make it clear by whatever means possible that theirs is a tenancy relationship. For example, on *Dr T’s* stationery a notation such as ‘solo practice’ should be sufficient to clarify the situation.

### Scenario 7: A patient, a spouse, a receptionist, an HCP

This 2001 case, *Alexander v Heise [2001] NSWSC 69,* took place in New South Wales, Australia. The actual names of the protagonists have been replaced to fit with the scenario. *C’s* spouse *S* telephoned *Dr D’s* clinic explaining *C’s* symptoms and requesting an urgent appointment for *C*. *Dr D’s* receptionist *R, is Dr D’s* spouse. *R* explained that there were no extended appointments available on the day. *R* booked an appointment for *C* 1 week later. During the intervening week, *C* died due to a ruptured berry aneurysm. *S* sought damages alleging:

- Defendants *Dr D* and *R* owed a duty of care to *C*.
- *Dr D* was vicariously liable for *R’s* breach of duty.
- *Dr D* breached his duty of care by not having sufficient protocols in place for *R* to follow in these matters.

The defendants argued that:

- there is no duty of care ‘on the part of a medical practitioner to attend upon a person who is sick, even in an emergency, if that person is one with whom the doctor is not and has never been in a professional relationship of doctor and patient’;
- the administrative staff of a medical practice did not owe a duty of care, and
- a doctor could not be vicariously liable for information known to his or her administrative staff.

Does *R* have duty of care to *C*? Does *Dr D* have a duty of care to *C* even though *C* never attended the clinic? Is *Dr D* vicariously liable for *R’s* alleged breach of their duty of care to C? Answer: Once C’s symptoms were known to *R* and an appointment made, both *Dr D* and *R* had a duty of care to C.

### Explanation

The NSW Supreme Court found that *R* was *Dr D’s* employee, hence *Dr D* was vicariously liable for *R’s* actions. The court found that once *C’s* symptoms were described to *R* and an appointment was made, *C* became a patient of the practice. It was held that a medical receptionist owes a duty of care to the patient to take reasonable care to “ensure that if he or she presents with a possible urgent medical condition, that patient is seen in a timely manner”.

The court decided that a general practitioner has ‘the responsibility to determine whether a patient requires urgent medical attention’ and then to ensure that the patients seeking appointments are properly prioritised. The court found that a medical receptionist owes a duty of care to the patient to ‘ensure that if he or she presents with a possible urgent medical condition, that patient is seen in a timely manner’. In effect a medical receptionist is triaging potential patients on the doctor’s behalf when an appointment request is made. Obviously, a doctor cannot triage potential patients seeking appointments. However, with proper policies, procedures and training in place, a receptionist’s index of concern would be sufficiently raised to realise that the doctor must be alerted to a patient requiring urgent attention and an immediate appointment made or other appropriate arrangements made for the patient's care.

The court found that neither *Dr D* nor *R* had breached their duties of care. *R* had not breached her duty because *S* did not convey a real sense of urgency when making the appointment thus *R* did not consider the matter urgent and scheduled an appointment for 1 week later, when the *Dr D* had a lighter evening schedule. *Dr D* did not breach his duty of care because he had protocols in place for dealing with patients presenting with urgent complaints and had provided sufficient informal training to ensure that the protocols were known to *R* [21].

### How to mitigate the risk

Seven scenarios have been presented demonstrating various ways a practitioner may be exposed to lawsuits. While it is impossible to have complete protection, many steps can be taken to mitigate the risk.

### Insurance

The best first step is to ensure your error and omissions insurance is up to date and that you have adequate limits. One policy covering all staff may not be adequate. The creation of well-managed working relationships that are properly documented are key to understanding where the risks might be. The extent of professional liability coverage can then be adequately reviewed, and additional coverage purchased if necessary because professional indemnity may not cover vicarious liability for clinical staff.

### General principles

Set high standards for your practice, and then make sure goals and procedures are followed. Act as a positive role model for business associates, stressing documentation and ethical behaviour. An overarching principle is captured in the adage: if it’s not in writing, it doesn’t exist, it never happened. This is worth remembering.

### Staffing

#### Hiring

Perform and document due diligence to ensure that only qualified staff who meet all educational and professional requirements for their position are engaged. Check all credentials and references to ensure truth and accuracy.

#### Contracts

In all instances where a principal engages the services of an associate, a written agreement setting out terms and conditions should be entered into. This may be an employment contract, a collaborative practice agreement or a locum agreement. A contract may be written, verbal or on a handshake. Verbal or handshake agreements are subject to the same contract principles that apply to written contracts. While a verbal contract or a handshake deal *may be* as enforceable as a written contract, they are difficult to prove if they breakdown. This makes a written contract always preferable to a verbal contract or a handshake deal.

#### Training

Properly train and teach all practice members their area of responsibility. A clearly documented articulation of this process is helpful.

Take the time necessary to teach staff all systems the practice has in place in order to deliver high quality care and prevent health care negligence or fraud at all levels. Staff should not stray into areas in which they are not trained, no matter how well intentioned their actions may be. Staff should make no decisions based on past ‘customary practice’. To ensure this, written guidelines will need to be in place. These are appropriately housed in a clinic practice manual detailing job description, chain of command, clinic policies and procedures. The clinic manual should be easily accessed, regularly reviewed and updated.

The greater the responsibility a position entails, the greater the risk it brings. In the case of the chiroractic assistant (CA)/receptionist, the more information that is relayed from the patient, the greater is the likelihood that ‘relevant information’ will either be missed or misinterpreted. Of necessity policies and procedures need to be in place for all persons likely to be making appointments to recognize and react appropriately to emergency situations.

While some activities appear as simple tasks, they may require knowledge of the potential risks, as well as reflective practice skills or ‘knowledge-in-action’ which is not easily reduced to rules and procedures. The more complex the task, the more care that must be taken in training. For example, generally, booking an appointment over the telephone is a simple task but not always. A telephone request for an appointment by a person telling the CA they have severe low back pain, a feeling of weakness and numbness in their legs is a potential risk situation. They may have cauda equina syndrome and require emergency decompression surgery. Failure by the CA to recognize this, and convey appropriate information to their employer and patient, may be negligent. Consideration should be given to all aspects of practice life including communications via emails which may be defamatory in nature. For example, in a dispute between staff [42].

Ensure that all clinic staff obtain continuing education credits in risk management annually. These implications extend to appropriate IT training and computer software that ensures data releases are appropriate and data breaches are prevented.

#### Supervision

Put in place appropriate and adequate supervision for all practice members. Attention should be given to patient encounter documentation, billing, and referrals, in addition to ways of recording the quality of these parameters. Insurance fraud is not uncommon and brings heavy penalties, both financial and custodial [43].

Develop a system to regularly review the work of practice members. Readily available and easily accessible open channels of communication that encourage frequent interaction with staff members, especially when a question arises involving patient care and/or potential health fraud issues should be in place. Stress to practice staff that any question or concern regarding error or fraud should be brought to your attention immediately.

#### Task delegation

Task Delegation is the assignment of authority to another person to carry out specific activities. It must be done on the basis of knowing that the person delegated is both qualified and competent to perform the task.

The issue for determination is whether the level of skill of the practice member was such that the risk of injury to the patient was foreseeable and significant such that any reasonable person would have taken precautions against the occurrence of such a risk. The same question could be anticipated of the person who delegated the tasks to the individual staff member in question.

#### Advertising

For practices utilizing genuine independent contractors, clear indications should be evident demonstrating the nature of the independent contractor relationship to avoid misleading the public.

Signs indicating this independence should be conspicuously placed and easily readable. This independence should also be evident in any marketing, advertising or office documents such as Informed Consent.

Consideration must be given to the impression clinic publications send to the public. This includes clinic newsletters, webpages, and social media pages. Phrases like ‘team member’, ‘a valuable member of the team’, ‘meet our doctors’ etcetera may mislead the public to reasonably believe that a genuine independent contractor acts in another capacity such as an employee with obvious legal implications.

Practices should also consider the use of a common logo as this implies a relationship which may not exist. Does the practice offer other services and how are these presented to patients? Is it in a manner that implies an employee relationship? For example, in Scenario 2, suppose the acupuncturist is a genuine independent contractor but is depicted as a member of ‘our team’ on the clinic website and displays the clinic logo on clothing along with a name badge with the clinic name on it. Would a member of the public reasonably believe this person was an employee of the clinic? Probably. The key question is: who chose the provider [44]? Did the entity engage the services of the provider? If so, the public will not be misled by the provider’s appearance and depiction. If the provider is a genuine independent contractor, this needs to be clearly conveyed to the public. This necessitates practice billing procedures, signage and stationery need to indicate the independence provider from the practice owner [45]. To this end ‘private practitioner’ may suffice.

#### Compliance requirements

Healthcare clinics have many compliance requirements. Develop, distribute, and implement written policies, procedures, and standards of conduct along with supporting documentation. This documentation should be reviewed regularly, at least yearly.

Example compliance questions to consider are:

1. Is the clinic constructed, arranged, and maintained to ensure access to and safety of patients?
2. Does the clinic provide adequate space for the provision of services?
3. Does the clinic comply with relevant occupational health and safety regulations?
4. Is all essential mechanical, electrical and patient-care equipment maintained in safe operating condition? How is this documented?
5. Are the premises clean and orderly?
6. Does the clinic have clear emergency procedures and staff training that assures the safety of patients if non-medical emergencies occur?
7. Are Exit signs are placed in appropriate locations?
8. Are emergency exits free from blockages?
9. Does the practice owner provide adequate services and equipment for their associates for patient care?
10. Does the principal participate in developing, executing, and periodically reviewing the clinic’s written policies with the associate member(s)?

## Conclusion

This paper has explored the duty of care in negligence and liability for breach of the duty of care in the context of a variety of practice arrangements where vicarious liability and non-delegable duties come into play. We examined the responsibility an employer bears for the negligent actions of employees or contractors. Among other duties, the employer is assumed to have diligently researched their agents’ credentials, licensure, and suitability to provide care.

Chiropractic care is most commonly located within a private practice context. This landscape is dynamic and is affected by emerging evidence, changing technology, societal and workplace expectations. In practice, as in life, everything is fine, until it isn’t. When things go “pear-shaped” it is important to know adequate protections are in place. Insurance policies, employment contracts, documented practice policies and procedures are critical components.

Oscar Wilde wrote “To expect the unexpected shows a thoroughly modern intellect.” But, as Monty Python humorously reminded us “nobody expects the Spanish Inquisition”. So where does that leave us? The “captain of the ship” may unexpectedly find themself legally responsible for another’s actions with dire financial consequences. Knowledge of VL and a timely practice review has the potential to minimize the unexpectedness, distress and financial loss of this unwanted event. This paper provides an overview of this area of law. Every effort has been made to ensure the paper’s accuracy. It is not intended to provide legal advice. Individual situations will differ, and the law may have changed since publication. Readers should consult with an experienced lawyer to understand current laws and how they may affect their particular circumstance.

## Data Availability

Not applicable.

## Abbreviations

ECC: Everywhere Chiropractic Clinic
HCP: Health care professional
NDD: Non-delegable duty
VL: Vicarious liability
CA: Chiropractic assistant
